# Incidence and predictors of virological failure among HIV infected children and adolescents on first-line antiretroviral therapy in East Shewa hospitals, Oromia Region, Ethiopia: A retrospective follow up study

**DOI:** 10.1371/journal.pone.0289095

**Published:** 2023-11-30

**Authors:** Netsanet Melkamu Abera, Tewodros Getaneh Alemu, Chilot Desta Agegnehu

**Affiliations:** 1 Department of Nursing, College of Medicine and Health Sciences, Dire Dawa University, Dire Dawa, Ethiopia; 2 Department of Pediatrics and Child Health Nursing, School of Nursing, College of Medicine and Health Sciences and Comprehensive Specialized Hospital, University of Gondar, Gondar, Northwest Ethiopia; 3 School of Nursing, College of Medicine and Health Sciences and Comprehensive specialized hospital, University of Gondar, Gondar, Ethiopia; Universitas Indonesia Fakultas Kedokteran, INDONESIA

## Abstract

**Introduction:**

Despite gains made from improved antiretroviral therapy coverage in resource limited countries, the occurrence of first line drug resistance remains a priority agenda. To reduce the emergence of resistant viruses, HIV viral load monitoring plays a critical role. However, many resource limited countries have difficulty of monitoring viral load due to economic constraints.There is also limited study regarding viral failure in developing countries. Therefore, this study aimed to assess the incidence and predictors of virological failure among HIV-infected children and adolescents on first-line ART Ethiopia, 2021.

**Methods:**

Institution based retrospective follow-up study was employed on 492 children and adolescents. Data were collected by trained nurses who have experience working in ART clinics. Data were entered using Epi-data version 4.6 and exported to Stata version 14 for analysis. The proportional hazard assumption was checked, and the Weibull regression was fitted. Cox-Snell residual was used to test the goodness of fit, and the appropriate model was selected by AIC. Finally, an AHR with a 95% CI was computed, and variables with a P-value < 0.05 in the multivariable analysis were taken as significant predictors of virological failure.

**Results:**

The overall incidence rate of virological failure was 4.2, (95% CI: 3.41, 5.22) per 1000 person-months of observation with 20,169 person-months follow-up time. In multivariable analysis living in rural area (AHR = 1.97, 95% CI: 1.15–3.36), poor adherence (AHR = 2.20, 95% CI: 1.24–3.91), lower CD4 Count <200 cells/mm3 (AHR = 2.57, 95% CI: 1.27–5.18) and 201–350 cells/mm3 (AHR = 2.44, 95% CI: 1.28–4.67) respectively, and recent OI (AHR = 4.60, 95% CI: 2.38–8.90) are significantly associated with virological failure.

**Conclusion:**

The incidence rate of virological failure was high. Living in a rural, poor adherence, lower CD4 count, and recent opportunistic infection were independent risk factors associated with virological failure. Hence, it is better to give priority to strengthening the focused evaluation of important variables and managing accordingly.

## Introduction

Virological failure is defined as a viral load above 1000 copies/ml based on two consecutive viral loads (VL) measurements, after 6 months of Antiretroviral Therapy (ART) initiation and with 3 months of enhanced adherence support following the first viral load test [1, 2].

Globally 38 million people are living with Human Immunodeficiency Virus (HIV), of which 25.8 million reside in Africa [3]. Particularly sub-Saharan Africa is home to 9 out of 10 HIV-infected children less than 15 years [4], and 83% of new adolescent HIV infections in 2019 [3]. According to global statistics in 2019, 1.7 million new HIV infections and 0.7 million deaths have been reported. Moreover, under-five children account for 60% of HIV-related deaths [5].

Although there are limited studies conducted on VF among children and adolescents in sub-Saharan Africa, in a study conducted in South Africa, the incidence of virological failure 3 years following initiation of ART was 19.2%.19.2% [6]. In addition, studies conducted in Rhode Island in the United States, United Kingdom and Ireland reported a VF incidence of 57% [7], 69% and 34% [8] respectively. Across-sectional studies conducted in resource-limited countries shows prevalence of VF ranges 11% to 66% [9–11]. In addition study conducted in Ghana states the three years probability of VF were 31% [12]. Furthermore a cross-sectional study conducted in Ethiopia shows the incidence of VF in pediatrics was 18.3% [13].

Maintaining long-term viral suppression among children and adolescents on ART is very challenging [14]. Their disease progression also very rapid with poor outcomes [2]. Furthermore incidence of VF is rising, but children receiving second-line regimens remain low compared to adults [15]. This indicates poor identification and switch of people failing first-line ART [16]. Failure to detect virological failure early and continuation of the failing regimen may result in the accumulation of resistant viruses leading to clinical deterioration and death [1, 17].

Different studies conducted have indicated that various factors were associated with virological failures such as being male [18], poor adherence [19], younger age [20], baseline CD4 count [21], clinical stage [11], and Nevirapine based regimen [22].

Despite gains made from improved antiretroviral therapy coverage in resource limited countries, the occurrence of first line drug resistance remains a priority agenda [23]. However compared to adults, a global coverage of ART in pediatrics is low (53%) [3, 5], their viral load status were also under-recognized issue that receives poor attention in the field of pediatrics and within HIV/AIDS programs [24]. Moreover, the majority of studies conducted in the resource-limited country are based on a combination of clinical, immunological, and virological [25–28]. However, currently both clinical and immunological failure criteria are insufficient in the diagnosis of treatment failure [29, 30]. The World Health Organization (WHO) also recommends VL monitoring as a preferred tool for diagnosing and confirming treatment failure [2, 31].

Different stakeholders are working collaboratively intending to create an HIV-free generation in 2030 [5]. The Global Fund strategy aims to reduce HIV incidence and mortality [2]. Sustainable Development Goal (SDG), also aims to end the HIV AIDS epidemic from being a public health threat by 2030 [32]. WHO and UNAIDS have set a goal of 90% by 2020 (90% of people living with HIV know their HIV status, access treatment and suppress their viral load), rising to 95% by 2030 [33]. Nevertheless, virological failure remains public health problem.

The majority of the studies conducted in resource-limited countries were based on a combination of immunological, clinical and virological failure. This would underestimate the incidence of treatment failure (TF) and leads to improper management of patients on ART. Therefore, this study aimed to determine the incidence and predictors of VF among children and adolescents on first-line ART in East Shewa, Oromia region Ethiopia, 2021.

## Method and materials

### Study design, period, and area

An institutionally based retrospective follow-up study was carried out. Data were collected from April 1–30, 2021 among HIV infected children and adolescents on ART between January 1, 2015, to December 31, 2019, in East Shewa, Oromia region, Ethiopia. The study was conducted in five Oromia regional public hospitals namely Adama college referral hospital, Bishoftu general hospital, Olenchity hospital, Batu hospital and Mojo hospital. HIV care and treatment clinic is one of the core parts of outpatient activities, all hospitals provide service to Adult and Pediatric ART. The ART case team in the hospital is composed of ART-trained physicians, ART-trained nurses, pharmacists, laboratory technicians, data clerks, and drug adherence counselors. Currently, the hospital serves more than 10thousand patients.

### Population, sample size determination, technique, and sampling procedure

The source population was all HIV-infected children and adolescents aged ≤ 19 years old on the first-line ART ever enrolled in ART clinics in East Shewa public hospitals and the study population was all HIV-infected children and adolescents aged ≤ 19 years old who had been on first-line ART for at least 9 months in East Shewa public hospitals from January 2015 to December 2019.

The sample size was determined by using a single population proportion formula with considerations of the following statistical assumption; confidence level 95%, the incidence of virological failure = 5.1% [34] (on a study conducted in the Tigray region) and 2% margin of error (D). Therefore, the minimum sample size was **465**. By considering incomplete patient records, 10% of the initial sample size was added and the final sample size was 511 [35].

The total sample size was proportionally allocated for every five Hospitals depending on their load of patients registered in the study period. To select study participants, a simple random technique was used in each hospital based on their allocation. Finally, 492 patient charts that fulfilled the inclusion criteria were included in the analysis.

### Variables of the study

The dependent variable was the incidence of virological failure, whereas the independent variables were socio-demographic characteristics of children and adolescents and caregivers (age, sex, residency, disclosure status, age of caregiver, relationship with caregiver, occupation, and marital status of the caregivers. Anti-retroviral medication-related characteristics adherence, ART regimen type, ART regimen change, isoniazid prophylaxis, and Co-trimoxazole prophylaxis. Clinical and immunological characteristics such as baseline nutritional status, baseline and recent WHO clinical stage, baseline CD4 count, and recent opportunistic infection.

### Definition of variables

#### Virological failure

Viral failure is defined as a viral load above 1000 copies/ml based on two consecutive viral loads (VL) measurements, after 6 months of ART initiation and with 3 months of enhanced adherence support following the first viral load test [1].

#### Survival time

Defined as the time in a month from the start of first-line ART treatment to the development of virological failure.

#### Event

Defined as patients who developed virological failure during the follow-up time.

#### Censored

Defined when the study participants lost, transferred out, died, and were free from the event during the follow-up time or end of the follow-up study.

#### Adherence to ART medications

Classified as good, fair, and poor according to the percentage of drug dosage calculated from the total monthly dose of ART drugs as follows: Good (equal to or greater than 95% or ≤ 3 doses were missed per month), Fair (85–94% or 4–9 doses were missed per month), or Poor (less than 85%) [1, 2].

#### Anemia

Was defined according to WHO criteria (hemoglobin <11 g/dl for children <5 years; hemoglobin <11.5 g/dl for children 5–11.99 years; hemoglobin <12 g/dl for children 12–14.99 years; hemoglobin <12 g/dl for females aged ≥ 15 years; hemoglobin <13 g/dl for males aged ≥ 15 years) [36].

### Data collection tool and procedure

Data were extracted by using an appropriate data extraction tool which was adapted in the English language from national HIV intake and follow-up care after checking the availability of variables in the HIV patient registration book. Three nurses who have experience working in ART clinics were selected for data extraction. The patient’s charts were retrieved by using the patient’s registration number from the database. Finally, charts that had a completion date of ART enrollment and date of VL measurement were selected and variables also were documented. To ensure the quality of data, data extraction tools were checked for the existence of variables in the registration format on the patient’s chart via a Preliminary chart review of 5% of the sample. Data collectors were nurses who had experience working in ART clinics. In addition, data collectors were given training for one day in each hospital before the start of data collection. Dara was extracted from April 1 to 30, 2021, from patient charts that fulfilled the inclusion criteria. The data retrieval process was closely monitored by the principal investigator and two supervisors. Besides, each data extraction tool was checked again immediately for its completeness. If there were any unfilled space happened during data collection time, the principal investigators were contacted by data collectors to take immediate corrective action.

### Data quality control, data processing, and analysis

The collected data was entered using EPI data version 4.6 statistical software and exported to Stata version 14 statistical software for further analysis. Descriptive statistics were carried out using tables and graphs. The Kaplan Meier failure curve method was used to estimate the time to virological failure. The Log-rank test was used to compare Survival curves between different categories of explanatory variables. Proportional hazard assumption was checked both graphically and using a Schoenfeld residual test which assesses the relationship between the scaled Schoenfeld residuals and time. The model of fitness was checked by Nelson- Aalen cumulative hazard rate relative to Cox-Snell residuals. The appropriate model for the data was selected based on Akaike Information Criterion (AIC), Bayesian Information Criterion (BIC), and log-likelihood (LL). The frailty model was taken into account random effect model for time-to-event data, by adding a frailty term to handle variations among hospitals. After the selection of our appropriate model, P-values ≤ 0. 25 in the bi-variable analysis were entered into the multivariable analysis and an adjusted hazard ratio (AHR) with a 95% confidence interval (CI) was designed as P-value < 0. 05 was considered as statistically significant.

### Ethical considerations

Ethical clearance was obtained from the ethical review committee of the school of nursing on behave of the institutional review board of the University of Gondar and a Permission letter was obtained from Adama medical college and referral hospitals, Bishoftu general hospitals, Olenchity hospitals, Batu hospital’s and Mojo hospital’s management and HIV care clinics focal person to use the secondary data for this study. The name or any other identifying information was not recorded on the questionnaire and all information taken from the chart was kept strictly confidential and in a safe place. An official ethical letter was received from the University of Gondar’s ethical review committee as well as the management of the hospitals where the study was done. As this study used secondary data, we do not have verbal or written consent.

## Results

### Description of study participants

**Fig 1:**
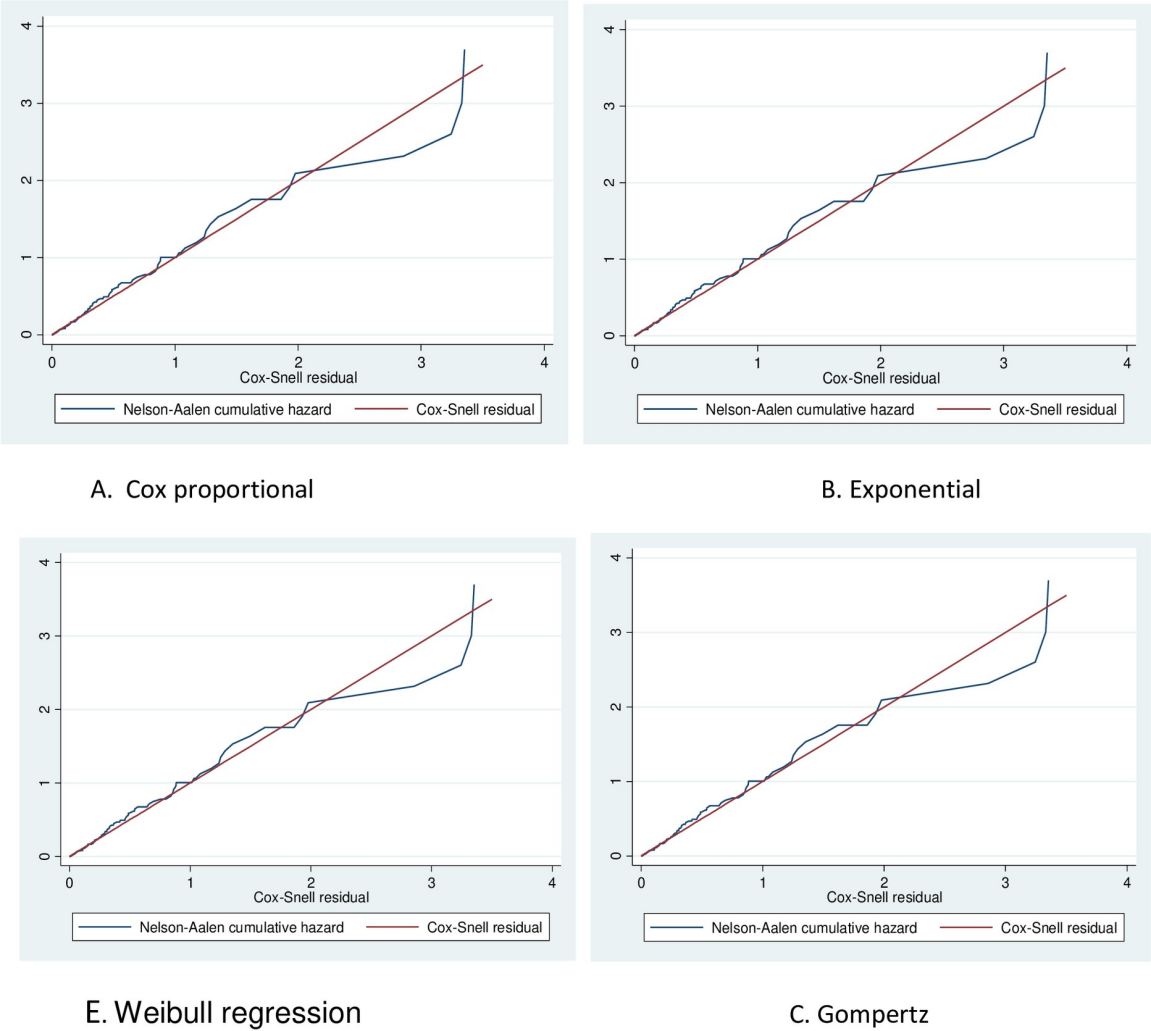
Plot of Nelsen-Aalen cumulative hazard function against Cox-Snell residual obtained by fitting Cox, exponential, Gompertz, and Weibull models for the virological failure of HIV/AIDS patients on first-line ART in public hospitals in East Shewa, Oromia region, Ethiopia, January 2015—December 2019.

A total of 511 medical charts were included of which 19 were excluded from a study due to missed charts and incomplete data, so a total of 492 charts were included in the analysis [Fig 1].

### Socio-demographic characteristics

The median age of the study participants at ART enrolment was 7.94 with an IQR of 7 and half of the study participants were female 50.2%. Three fourth 377(76.63%) of the study participant were from urban dwellers. Regarding the relationship of caregivers of the study participants 410(83.33%) were parents and 352(71.54%) of the caregivers of the study, participants belonged to the < 40 years age group. Regarding occupational status, 187(38.0%) of the caregivers were housewives [Table 1].

**Table 1:** Baseline socio-demographic characteristics of HIV-infected children and adolescents on first-line ART in Oromia regions East Shewa hospitals, Ethiopia from September 2015 to December 2019 (N = 492).

Variables	Category	Frequency	Percent
Age	<5	132	26.83
	5–10	172	34.96
	10–14	151	30.69
	15–19	37	7.52
Sex	Male	247	50.20
	Female	245	49.80
Residence	Urban	377	76.63
	Rural	115	23.37
Age of the caregivers	< 40	352	71.54
	≥ 40	140	28.46
Relationship of caregivers for the child	Parent	410	83.33
	Relatives	47	9.55
	Guardians/neighbors	14	2.85
	Others	21	4.27
Marital status of the caregivers	Never married	17	3.46
	Married	375	76.22
	Widowed	72	14.63
	Divorced	28	5.69
Occupation of caregivers	Unemployed	64	13.01
	Governmental	111	22.56
	Nongovernment	8	1.63
	Housewife	187	38.01
	Private	83	16.87
	Farmer	15	3.05
	Daily laborer	24	4.88

### Baseline clinical and immunological characteristics

The majority of study participants were assigned to WHO stage one 284(57.72%), and had CD4 above 500 copies per cell 310 (63.01%). Diarrhea is the most common opportunistic infection 44(21.15%). Regarding baseline developmental and functional status, most of the study participants had appropriate 120(90.91%) and working baseline developmental status 306 (85.0%), respectively [Table 2].

**Table 2:** Baseline clinical and immunological characteristics of HIV-infected children and adolescents on first-line ART in Oromia regions East Shewa hospitals, Ethiopia from September 2015 to December 2019 (N = 492).

Variables	Category	Frequency	Percent
WHO Clinical stage	Stage I	284	57.72
	Stage II	20	4.07
	Stage III	182	36.99
	Stage IV	6	1.22
CD4 Count at initiation (cells/mm3)	<200	55	11.18
	200–350	75	15.24
	351–500	52	10.57
	≥ 500	310	63.01
Opportunistic infection at baseline	TB	44	21.15
	Fever >1 month	26	12.50
	Pneumonia	38	18.36
	Diarrhea	65	31.25
	Candidiasis	9	4.33
	URTIs	20	9.62
	Others	6	2.88
Baseline developmental status for <5 years children	Appropriate	120	90.91
	Delayed	12	9.09
Baseline functional status for ≤ 5 years children	Working	306	85.00
	Ambulatory	52	14.44
	Bedridden	2	0.56

### ART and medication-related factors

More than half of study participants 272 (55.28%) have initiated NVP-based NNRTI regimen. About 24 (4.88%) of the study participants had a history of use of PMTCT (Prevention of Maternal to Child Transmission) service. Of the total 492 patients, 443 (90.04%) and 425 (86.38%) had been placed on cotrimoxazole and isoniazid prophylaxis respectively. Among the total, 428 (86.99%) of the study participant had good and fair adherence. Regarding disclosure status, 198(40.24%) of children and adolescents disclosed their HIV status [Table 3].

**Table 3:** Follow-up data on factors related to ART and other medications of HIV-infected children and adolescents on first-line ART in Oromia regions east Shewa hospitals, Ethiopia from September 2015 to December 2019 (N = 492).

Variables	Category	Frequency	Percent
Baseline NNRTI regimen	NVP	272	55.28
	EFV	141	28.66
	PI	54	10.98
	DTI	14	2.85
	Others	11	2.24
ARV prophylaxis for PMTCT	Yes	24	4.88
	No	468	95.12
Baseline opportunistic Infections	Yes	208	42.28
	No	284	57.72
Co-trimoxazole prophylaxis	Yes	443	90.04
	No	49	9.96
Isoniazid prophylaxis	Yes	347	70.53
	No	145	29.47
Adherence	Good	425	86.38
	Fair/poor	67	13.62
Disclosure to children	Disclosed	198	40.24
	Not disclosed	294	59.76
Disclose to others	Yes	420	85.37
	No	72	14.63

### Reasons for ART regimens change

**Fig 2:**
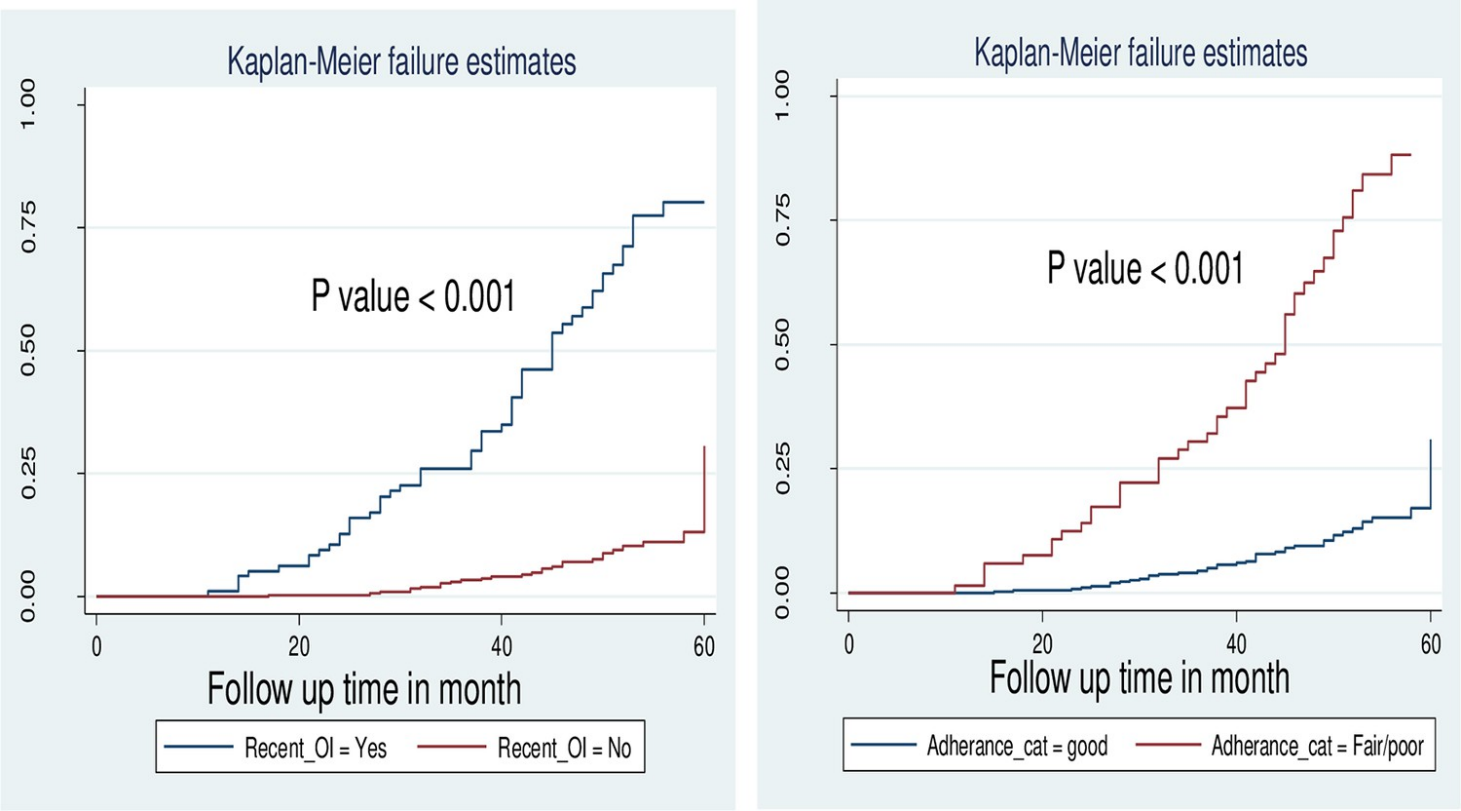
Kaplan failure hazard curve by (A) recent opportunistic infection (B) Adherence status of virological failure among HIV infected children and adolescents on first-line anti-retroviral therapy regimen at public hospitals in East Shewa, Oromia region, Ethiopia, January 2015 to December 2019 (N = 492).

In those patients with antiretroviral regimen changes, more than half 221 (62.78%) were changed due to ART stock out, 668 (18.18%) due to ART toxicity and 59 (16.76%) due to virological failure [Fig 2].

### The incidence rate of virological failure

**Fig 3:**
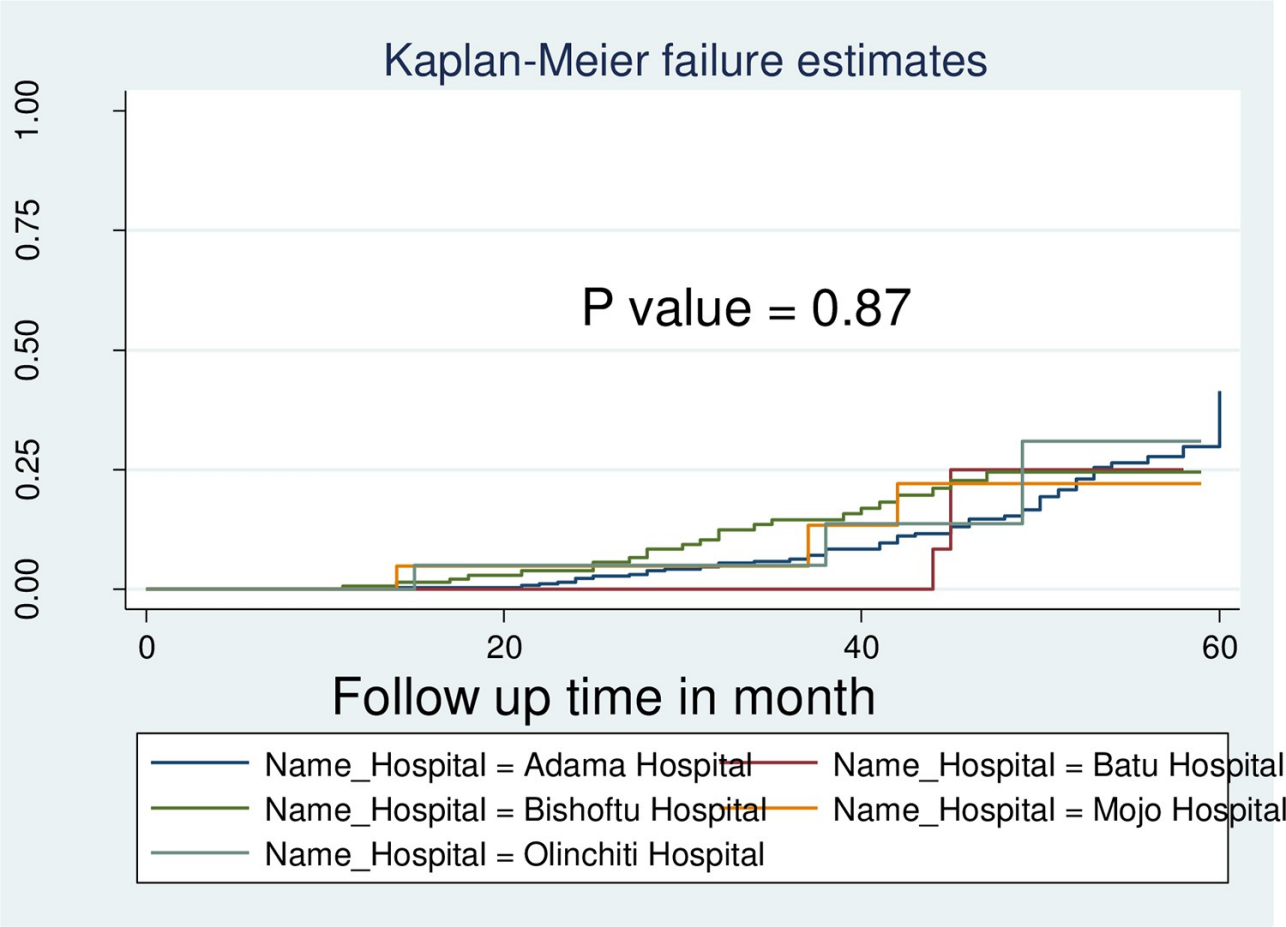
Kaplan Meier failure curve by hospitals among HIV infected children’s and adolescents on first-line antiretroviral therapy regimen at public hospitals in East Shewa, Oromia region, Ethiopia, January 2015 to December 2019 (N = 492).

Four hundred ninety-two children and adolescent HIV patients were followed for different periods for a total of 20,169 Person Months (PM) observations. The study participants were followed for a minimum of 10 months and a maximum of 60 months The incidence rate of first-line ART virological failure was 4.2 per1000 follow-up months (95% CI: 3.41, 5.22). The overall IR of virological failure at Adama, Batu, Bishoftu, Mojo, and Welenchiti hospitals were 4.23 (95% CI, 3.23–5.54), 3.75 (95%CI, 1.21–11.6), 4.36 (95%CI, 2.90–6.57), 3.77(95%CI, 1.21–11.7) and 3.98(95%CI, 1.28–12.3) cases per 1000PM observations, respectively [Fig 3].

### Predictors of virological failure

**Fig 4:**
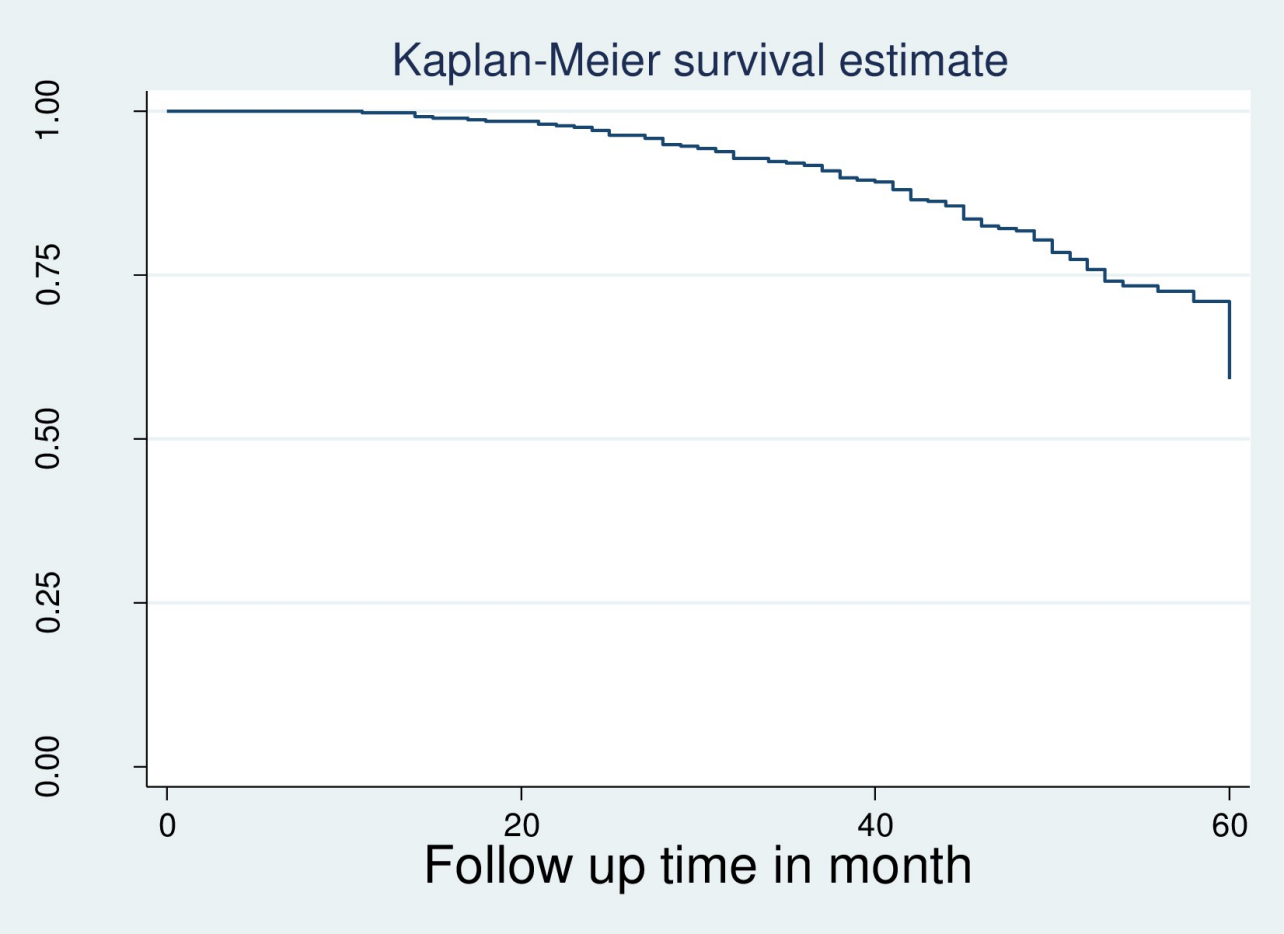
Kaplan Meier survival estimate of virological failure among HIV infected children and adolescents on first line ART in East Shewa Oromia region, Ethiopia from January 2015 to December 2019 (N = 492).

The Kaplan Meier failure function and log-rank test were used to show differences in survival experiences among different groups of categorical variables at baseline. Both the estimated survival curve and the log-rank tests showed that there was no overall difference among the survival curves of the hospitals (Log-rank Chi-square chi2 = 1.22 Pr> chi2 = 0.8755) [Fig 4].

**Fig 5:**
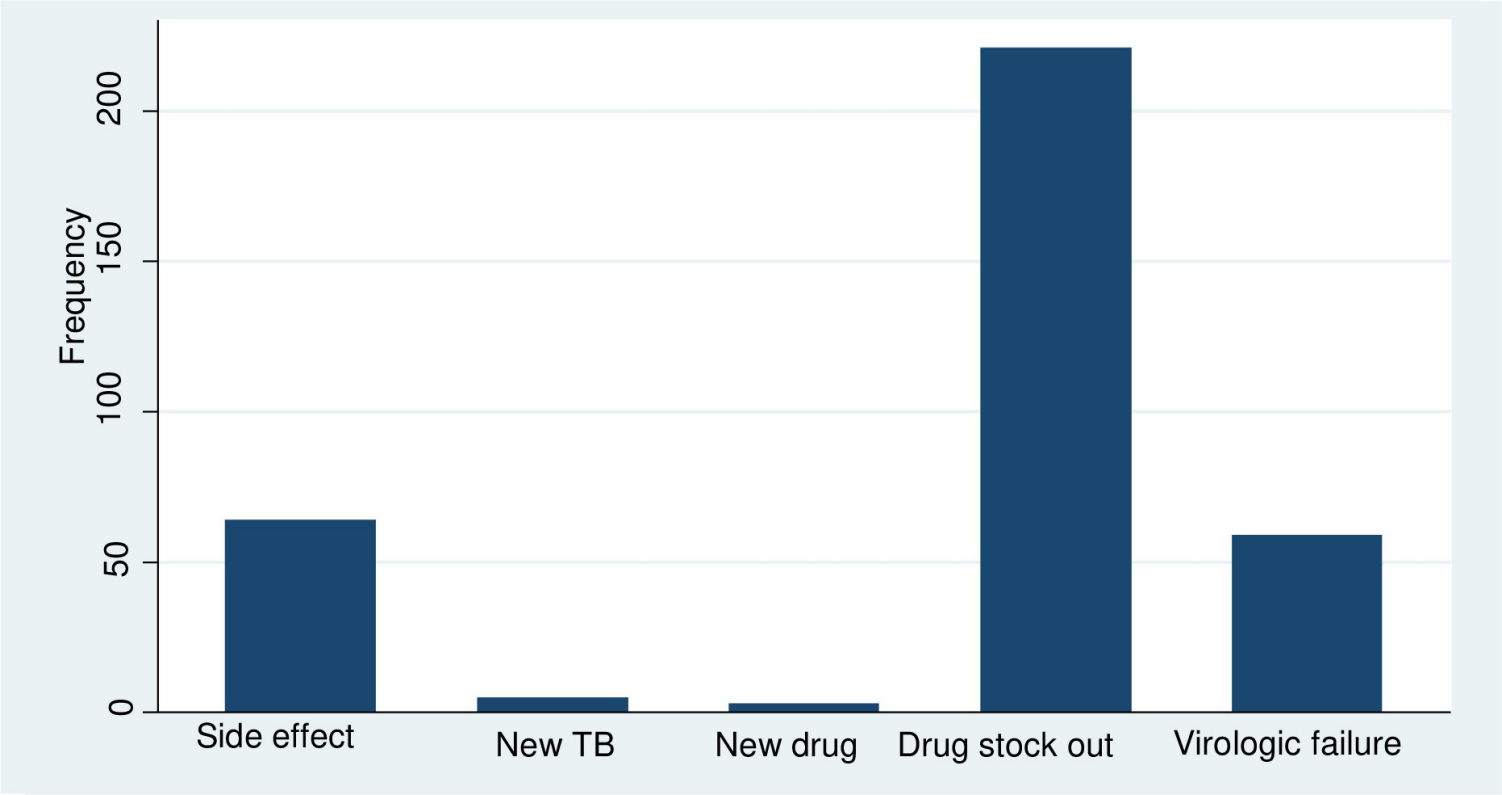
Reasons of ART regimens change among HIV infected children and adolescents on first-line ART in Oromia regions East Shewa hospitals, Ethiopia from September 2015 to December 2019 (N = 492).

In the case of the long rank test without adjusting other covariates, there were significant variations between the presence of recent opportunistic infection and not having a recent opportunistic infection (P<0.001) and in those who were in good and fair/poor adherence (P<0.001) [Fig 5].

### Assessing the proportional hazard assumption

**Fig 6:**
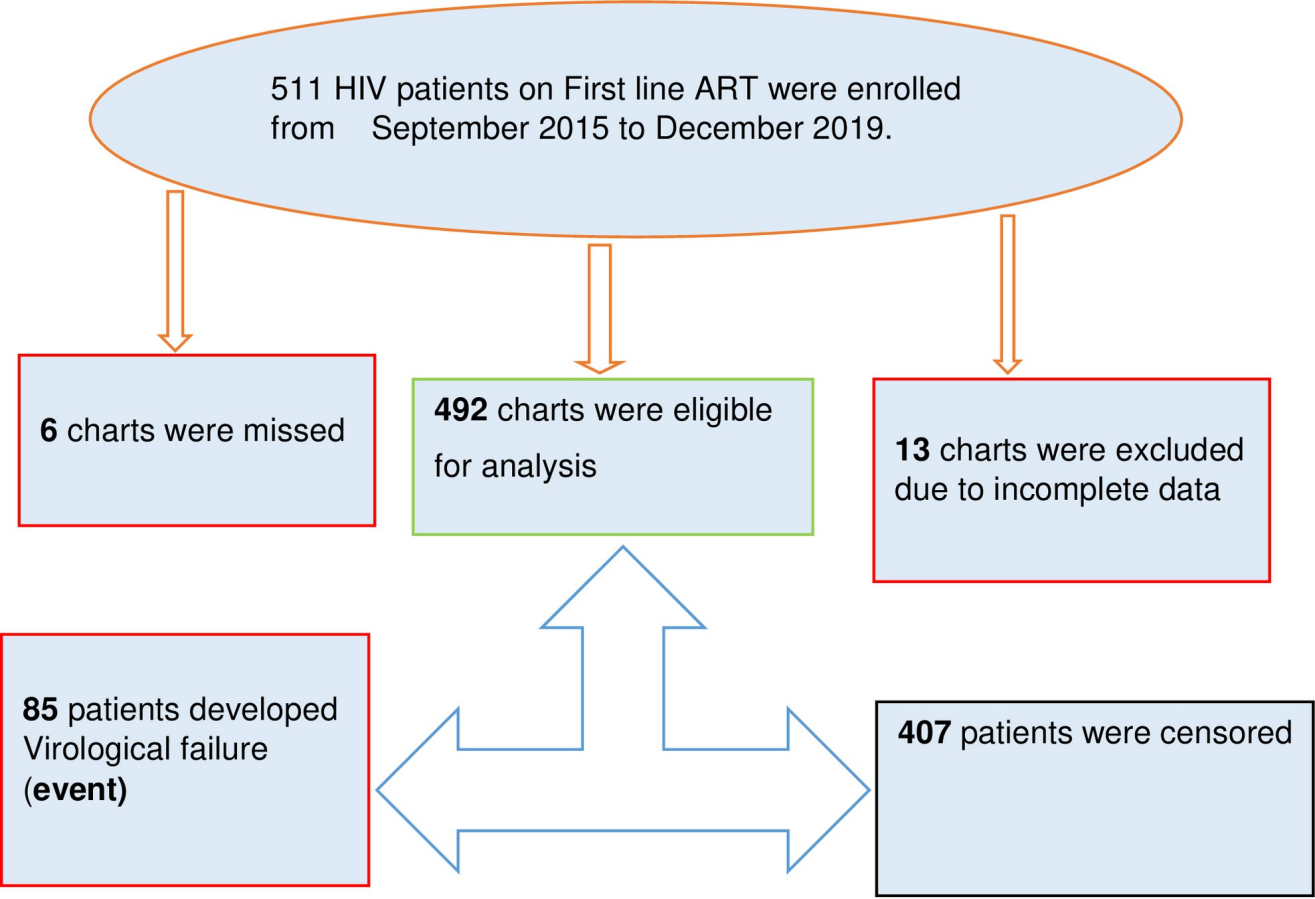
Flow chart showing a selection of HIV infected children and adolescents on first-line ART in public hospitals in East Shewa, Oromia region Ethiopia, January 2015—December 2019.

According to the Schoenfeld global test, the overall full model satisfies the proportional hazard assumption which states that the risk of failure of the study subjects must be the same no matter how long they are followed. (Chi-square = 10.01, p-value = 0.8188) [Fig 6].

### Model comparison

After the proportional hazard assumption was checked, both semi-parametric and parametric proportional hazard models were fitted to estimate the survival time to virological failure and identify its predictors among HIV patients on first-line ART. Information criteria (AIC, BIC) and log-likelihood were used to select the most parsimonious models for the data set.

Based on this, the Weibull regression with the (**AIC = 313.15, BIC = 405.32)** model was more efficient than Cox proportional hazard and other parametric models. On the other hand, the frailty effect by treatment hospitals was not a statistically significant variance between individuals among hospitals and also among individuals [Table 4].

**Table 4:** Summary of model comparison among the Cox proportional hazard model, parametric Cox- Regression models, and frailty models using AIC, BIC LR criteria.

Model	Baseline Hazard	Frailty	Variance	AIC	BIC	Log-likelihood
Cox regression	Unspecific			784.91	847.89	-377.45
**Weibull regression**	**Weibull**			**243.94**	**315.31**	**-104.97**
Shared frailty (Hospital)	Weibull	Gamma	p = (1.00)	245.94	321.51	-104.97
Univariate frailty (Hospital)	Weibull	Inv Gaussian	p = (1.00)	245.94	321.51	-104.97
Exponential	Exponential			368.20	435.38	-168.10
Gompertz	Gompertz			244.66	316.03	-105.33
Log logistic regression	Log logistic			245.87	317.25	-105.93
Lognormal regression	Log normal			245.26	316.63	-105.63

The Cox- Snell residuals versus Nelson-Aalen cumulative hazard function were obtained by fitting the Cox, Weibull, Gompertz, lognormal, log-logistic, and exponential models to the data. It can be seen that the plot of the Nelson-Aalen cumulative hazard function against the Cox-Snell residuals has a linear pattern making a straight line through the origin of the Weibull model when compared to cox, Gompertz, lognormal, log-logistic, and exponential models. This suggests that the Weibull regression model provided the appropriate fit for this data set [Fig 6].

### Model diagnosis

After the proportional hazard assumption was checked [Table 4], both semi-parametric and parametric proportional hazard models were fitted to estimate the survival time to virological failure and identify its predictors among HIV patients on first-line ART. Information criteria (AIC, BIC) and log-likelihood were used to select the most parsimonious models for the data set. Based on this, the Weibull regression with the (AIC = 243.94,) model was more efficient than Cox proportional hazard and other parametric models. On the other hand, the frailty effect by treatment hospitals was not a statistically significant variance between individuals among hospitals [Fig 5] and also among individuals [Table 4].

### Bivariable and multivariable analysis

In the Bi-variable Weibull proportional hazard model, sex of children, the residence of the child, marital of status caregivers, occupation of caregivers, disclosure to others, disclosure to the child, PMTCT, past OI, ART regimen change, adherence, recent OI, baseline hemoglobin, baseline CD4 count, baseline WHO count and recent WHO count were significantly associated with the incidence of Virological failure.

However, in multivariable analysis residence of children, poor adherence, lower baseline CD4 count, and recent OI were significantly associated with virological failure [Table 5].

**Table 5:** Bi-variable and multivariable analysis using the Weibull Cox regression model for predictors of first-line ART failure of HIV-infected children and adolescents in public hospitals in East Shewa, Oromia region, Ethiopia, January 2015–December 2019 (N = 492).

	Category	Status		Crude HR (95% CI)	Adjusted HR (95% CI)
		Event	Censored		
Sex of child	Male	47	200	1.33(0.87–2.05)	1.25(0.78–2.02)
	Female	38	207	1	1
Residence of child	Urban	50	322	1	1
	Rural	35	85	2.27 (1.45–3.55)	**1.97(1.15–3.36)** *
Marital status of the caregiver	Never married	1	16	1	1
	Married	57	318	3.60(0.49–26.07)	3.49(0.40–29.91)
	Widowed	18	54	6.69(0.89–50.18)	3.08(0.35–26.79)
	Divorced	1	27	6.74(0.85–53.28)	4.88(0.49–48.11)
Occupation of caregiver	Unemployed	19	45	1	1
	Governmental	21	90	0.48(0.26–0.90)	0.82(0.39–1.72)
	Nongovernment	3	5	1.50(0.44–5.08)	2.84(0.71–11.39)
	Housewife	21	166	0.31(0.17–0.59)	0.81(0.35–1.86)
	Private	13	70	0.51(0.25–1.05)	0.88(0.36–2.11)
	Farmer	4	11	0.81(0.27–2.40)	1.50(0.39–5.77)
	Daily laborer	4	20	0.46(0.15–1.36)	0.72(0.20–2.52)
Disclosure to children	Disclosed	27	171	1	1
	Not disclosed	58	236	1.65(1.04–2.60)	1.65(0.97–2.78)
Disclose to others	Yes	58	362	1	1
	No	27	45	2.96(1.88–4.68)	1.68(0.89–3.15)
PMTCT	Yes	6	18	2.03(0.88–4.67)	2.49(0.93–6.65)
	No	76	392	1	1
Past OI	Yes	64	144	1	1
	No	21	263	3.82 (2.33–6.26)	1.18(0 .26–5.22)
ART regimen changed	Yes	79	273		1.40(0 .58–3.38)
	No	6	134	1	1
Adherence	Good	40	385	1	1
	Poor/fair	45	22	8.98(5.85–13.79)	**2.20 (1.24–3.91)** *
Baseline CD4 Count (cells/mm3)	<200	23	32	5.21(2.94–9.23)	**2.57(1.27–5.18)** *
	200–350	24	51	3.92 (2.22–6.90)	**2.44(1.28–4.67)** *
	351–500	14	38	2.85 (1.47–5.52)	1.70 (0.82–3.51)
	> 500	24	286	1	1
Hemoglobin level	Anemic	19	26	3.52 (2.11–5.88)	1.24(0.74–2.06)
	Not anemic	66	381	1	1
Recent OI	Yes	58	40	11.85(7.48–18.75)	**4.61(2.38–8.90)** *
	No	27	367	1	1
Baseline WHO count	Stage I/II	24	280	1	1
	Stage III/IV	61	127	3.68 (2.29–5.90)	1.71(0.42–7.00)
Recent WHO stage	Stage T1/T2	53	397	1	1
	Stage T3/T4	32	10	11.34(7.26–17.72)	1.69(0 .89–3.20

* p-value <0.05 statistically significant

Keeping out other variables constant, the hazard of developing virological failure among children and adolescents living in rural areas was 1.97 times (AHR = 1.97, 95%CI: 1.15–3.36) more likely than those who were living in urban.

The hazard of developing virological failure in patients that had poor adherence 2.20 times (AHR = 2.20, 95% CI: 1.24–3.91) is more likely than in patients with good and fair adherence, keeping all other variables constant.

Regarding CD4 count keeping other variables constant, hazard of developing virological failure in patients with baseline CD4 count (<200 cells/mm3) and (200–350 cells/mm3) were 2.5 (AHR = 2.57, 95%CI: 1.27–5.18) and 2.4 times (AHR = 2.44, 95%CI: 1.28–4.67) more likely than > 500 cells/mm3 CD4 count respectively.

Moreover, for those children and adolescents who had recent infections keeping other variables constant, the hazard of developing virological failure 4.6 times (AHR = 4.61, 95% CI: 2.38–8.90) was more likely than no recent infection.

## Discussion

The overall incidence rate of virological failure in this study was 4.2 (95% CI: 3.41, 5.22) per 1000 person-months of observation. The result is in line with a study conducted in southern Ethiopia, Tigray region and Bahir Dar, with an incidence rate of 4.97 [21], 5.1 [34, 37] respectively. This similarity could be due to a similar period of study and cut-off point that had been used to define virological failure. In addition, the HIV/AIDS prevention and control program of Ethiopia has recently given special attention to people living with HIV/AIDS to meet 90% of targets as a national strategy may also have an impact [11].

However, the proportion of virological failure 17.2 (95% CI: 14.17–20.88) in our study was lower than studies conducted in Kenya (28%) [24], Cameroon 53% [18], Uganda 38% [38] and Bahir Dar (34%) [26]. This difference may be due to the difference in the definition of virological failure used, the cut-off point to define virological failure, and the difference in inclusion criteria and study year. A study conducted in Kenya and Cameroon defines virological failure as the presence of detectable virus in plasma greater than 500 copies/ml and 200 copies/ml respectively [18, 24]. Comparing our study, the difference in cutoff point to define virological failure, which was two measures of a viral load greater than 1000 copies/ml, would increase the number of virological failures in Kenya and Cameroon [14]. Whereas, a study conducted in Bahir Dar and Uganda define virological failure as a single rise in viral load measure above 1000 copies/ml, which would increase the number of virological failures in this study [26, 38]. Moreover, studies conducted in Bihar Dar include those children whose only viral load was requested into the laboratory within the study period (Each child sample was considered as a study unit and Children with complete requisition and plasma samples were used as inclusion criteria). In addition, in a study conducted in Uganda, death after at least 6 months of treatment was considered an outcome (virological failure), and this may further increase the number of virological failures. In addition, the difference in the study year 2011 vs 2019 and the study population (including only those under 12 years) may also be another factor for their difference [11]. This could be explained by the recent scaled-up use of viral load test for monitoring the effectiveness of ART in recent years.

This result was also higher than the study conducted in Jimma (11%) [11], the difference might be due to the difference in adherence and study population. Study participants in Jimma had better adherence than in our study area (92.1% vs 86.38%) [11]. As adherence was a significant factor in the occurrence of virological failure, the difference in adherence might be a factor for the lower occurrence of virological failure in Jimma. In addition, the difference in study design was cross-sectional vs follow-up study. Being a follow-up study gives you the chance to distinguish between those who developed VF and those who never reached suppression [14].

Furthermore, the difference in study population reflects the inclusion in Jimma of children less than 15 years may also be a factors.

In this study children and adolescents living in rural areas had HR of 1.97 times higher than those living in urban. This result is consistent with a study conducted in South Africa and Southern Ethiopia [39, 40]. This can be explained by being in rural areas will have an impact on long transport and time, lack of transportation, poor condition of roads, and transport costs imposed on patients when individual visits to a health center site distant from rural homesteads [14]. This may lead to poor adherence to medication and this in turn increases susceptibility to virological failure [39]. In addition, those living in the urban area may closely monitor their follow-up and may have more information regarding HIV/AIDS.

Regarding adherence, the present study showed that the AHR of children and adolescents who had poor/fair adherence to ART regimens were 2.20 times higher hazard of experiencing first-line virological failure compared to their counterparts. This result is consistent with studies conducted in Uganda [19, 38], Kenya [41], southern Ethiopia [21], central Oromia [42], Cameron [18], and Tanzania [43]. This is due to optimal adherence required to suppress virological load and improve clinical as well as immunology outcomes [34]. ART is recommended for HIV/AIDS patients to suppress viral load, maintain high immunity, and prolong survival. However, the success of ART depends on adherence to the treatment regimen [1]. Poor adherence decreases drug effectiveness, which declines immunity this will in turn increase the risk of opportunistic infection and drug resistance [37]. This in turn increases rates of viral replication, high rates of CD4 destruction, accumulations of resistant viruses, and faster rates of disease progression. This overall results in virological failure.

According to this study, baseline CD4 counts of (<200 cells/mm3) and (200–350 cells/mm3) are 2.5 and 2.4 times a higher hazard of developing virological failure compared to patients with more than 500 cells/mm3 CD4 count, respectively. This result is in line with a study conducted in Cameroon [18], Tanzania [20], and central Oromia [42]. This could be explained by a lower CD4 count will increase the risk of opportunistic infection which will further complicate treatment outcomes [25]. HIV/AIDS is a disease that decreases immunity and increases susceptibility to opportunistic infection. So, having low CD4 counts below a threshold at baseline increases the risk of opportunistic infection. This, in turn, increases the pill’s burden, side effect, and drug-drug interaction on child and adolescents, this overall results decrease in their adherence to the treatment regimen and increase the likelihood of virological failure.

Other predictors of virological failure in this study were recent opportunistic infection, those children and adolescents with recent opportunistic infection were 4.6 times more hazard of failure than those who had no recent opportunistic infection. This is consistent with a study conducted Bihar Dar [37]. This can be explained by children and adolescents with recent infection increasing the risk of concomitant opportunistic infection which in turn lowers their immunity and decrease their adherence to medication which further complicates the outcome [37]. In addition, those children and adolescents with recent opportunistic infections may have a severe disease like TB, which will increase drug burden and side effects, and decrease drug efficiency. This in turn results in virological failure.

### Limitation of the study

Since the design was retrospective, the study was unable to exhaustively explore all predictor variables that may have an effect on the incidence of virological failures such as socioeconomic status of the caregiver, educational status, and some laboratory assessments.

## Conclusions

The incidence of virological failure among children and adolescents on first-line ART at East Shewa public hospitals was found to be high. Living in a rural, lower CD4 count (< = 200 cells/mm3 and 201–350 cells/mm3), poor adherence, and recent opportunistic infection at baseline were found to be predictors of first-line ART virological failure. Therefore, it is better to give priority to strengthening the focused evaluation on important variables and managing accordingly.

## List of abbreviations

3TC: Lamivudine
ABC: Abacavi
AHR: Adjusted hazard ratio
AIC: Akaike Information Criterion
AIDS: Acquired Immune Deficiency Syndrome
ART: Antiretroviral Therapy
AZT: Zidovudine
BIC: Bayesian Information Criterion
CD4: Cluster Difference 4
D4T: Stavudine
CHR: Crude hazard ratio
HIV: Human Immune virus IR: Incidence Rate
LL: log-likelihood ratio
NVP: Nevirapine
PY: person year
SDG: Sustainable Development Goal
TDF: Tenofovi
TF: Treatment Failure
UNAIDS: United Nations program on AIDS
VF: Virological Failure
VL: Viral Load
WHO: World Health Organizations
